# Supplementary material for: Modeling glioblastoma heterogeneity as a dynamic network of cell states
Source: Mol Syst Biol. 2021 Sep 16;17(9):e10105. doi: 10.15252/msb.202010105 (PMC8444284; doi:10.15252/msb.202010105)
Supplement: Supplementary file 6 — Source Data for Figure 5 [file MSB-17-e10105-s004.zip › Figure5A_sourcedata/GSEA_3071/hallmarks_stateVI.GseaPreranked.1623742557031/HALLMARK_UNFOLDED_PROTEIN_RESPONSE.html]

Details for gene set HALLMARK\_UNFOLDED\_PROTEIN\_RESPONSE[GSEA]

|  || Dataset | state63071 |
| Phenotype | NoPhenotypeAvailable |
| Upregulated in class | na\_pos |
| GeneSet | HALLMARK\_UNFOLDED\_PROTEIN\_RESPONSE |
| Enrichment Score (ES) | 0.4143024 |
| Normalized Enrichment Score (NES) | 2.3360586 |
| Nominal p-value | 0.0 |
| FDR q-value | 0.0 |
| FWER p-Value | 0.0 |
Table: GSEA Results Summary

  

Fig 1: Enrichment plot: HALLMARK\_UNFOLDED\_PROTEIN\_RESPONSE      
 Profile of the Running ES Score & Positions of GeneSet Members on the Rank Ordered List

  

| PROBE | GENE SYMBOL | GENE\_TITLE | RANK IN GENE LIST | RANK METRIC SCORE | RUNNING ES | CORE ENRICHMENT || 1 | NPM1 |  |  | 114 | 0.717 | 0.0188 | Yes |
| 2 | NABP1 |  |  | 186 | 0.609 | 0.0419 | Yes |
| 3 | EIF2S1 |  |  | 287 | 0.547 | 0.0525 | Yes |
| 4 | EIF4A1 |  |  | 329 | 0.523 | 0.0779 | Yes |
| 5 | XBP1 |  |  | 389 | 0.494 | 0.0963 | Yes |
| 6 | HSPA5 |  |  | 414 | 0.484 | 0.1237 | Yes |
| 7 | ATF3 |  |  | 416 | 0.483 | 0.1573 | Yes |
| 8 | DKC1 |  |  | 430 | 0.478 | 0.1873 | Yes |
| 9 | DDX10 |  |  | 513 | 0.452 | 0.1963 | Yes |
| 10 | IMP3 |  |  | 667 | 0.408 | 0.1825 | Yes |
| 11 | NOP14 |  |  | 701 | 0.396 | 0.2012 | Yes |
| 12 | SDAD1 |  |  | 724 | 0.392 | 0.2226 | Yes |
| 13 | FKBP14 |  |  | 813 | 0.374 | 0.2245 | Yes |
| 14 | HERPUD1 |  |  | 830 | 0.371 | 0.2461 | Yes |
| 15 | RRP9 |  |  | 872 | 0.365 | 0.2604 | Yes |
| 16 | EXOSC1 |  |  | 887 | 0.362 | 0.2820 | Yes |
| 17 | SHC1 |  |  | 913 | 0.358 | 0.3002 | Yes |
| 18 | YIF1A |  |  | 964 | 0.351 | 0.3110 | Yes |
| 19 | SLC7A5 |  |  | 989 | 0.348 | 0.3287 | Yes |
| 20 | BAG3 |  |  | 1034 | 0.339 | 0.3404 | Yes |
| 21 | HSPA9 |  |  | 1087 | 0.333 | 0.3493 | Yes |
| 22 | EXOSC5 |  |  | 1192 | 0.317 | 0.3427 | Yes |
| 23 | PREB |  |  | 1229 | 0.312 | 0.3546 | Yes |
| 24 | ATF4 |  |  | 1371 | 0.295 | 0.3362 | Yes |
| 25 | GOSR2 |  |  | 1376 | 0.294 | 0.3558 | Yes |
| 26 | CEBPB |  |  | 1389 | 0.293 | 0.3730 | Yes |
| 27 | SERP1 |  |  | 1428 | 0.288 | 0.3827 | Yes |
| 28 | RPS14 |  |  | 1446 | 0.286 | 0.3980 | Yes |
| 29 | POP4 |  |  | 1464 | 0.283 | 0.4132 | Yes |
| 30 | NOP56 |  |  | 1531 | 0.276 | 0.4143 | Yes |
| 31 | NHP2 |  |  | 1656 | 0.264 | 0.3984 | No |
| 32 | NOLC1 |  |  | 1699 | 0.260 | 0.4050 | No |
| 33 | SEC31A |  |  | 1741 | 0.257 | 0.4117 | No |
| 34 | EIF4A2 |  |  | 1904 | -0.264 | 0.3852 | No |
| 35 | VEGFA |  |  | 2104 | -0.298 | 0.3509 | No |
| 36 | FUS |  |  | 2845 | -0.474 | 0.1788 | No |
| 37 | DCP2 |  |  | 3100 | -0.585 | 0.1494 | No |
Table: GSEA details [plain text format]

  

Fig 2: HALLMARK\_UNFOLDED\_PROTEIN\_RESPONSE: Random ES distribution      
 Gene set null distribution of ES for **HALLMARK\_UNFOLDED\_PROTEIN\_RESPONSE**
